# Collaborative Learning Communities With Medical Students as Teachers

**DOI:** 10.1177/23821205231183878

**Published:** 2023-06-20

**Authors:** Chenyi Yang, Konnor Davis, Michael Head, Nolan A. Huck, Tyler Irani, Andrew Ovakimyan, Aaron Frank, Andrew Cuyegkeng, Lauren Stokes

**Affiliations:** School of Medicine, University of California, Irvine, CA, USA

**Keywords:** medical education, peer assisted learning, near peer tutoring, medical student success

## Abstract

In recent years, peer-assisted learning has emerged as a new and effective medical education modality. Near-peer tutoring utilizes a senior student serving as an instructor to a junior student. In 2019, the University of California, Irvine, School of Medicine (UCISOM) implemented a near-peer tutoring model beginning with first-year anatomy and physiology curricula. Following a successful pilot program, UCISOM launched a full-fledged near-peer tutoring program in 2020 named Collaborative Learning Communities (CLC) with Medical Students as Teachers. The rollout of CLC occurred in phases. In 2020, second-year medical students led the program for first-year students; in 2021, an additional program was led by third-year medical students for second-year students; in 2022, the program expanded to third-year medical students led by fourth-year students. Each program serves the unique learning needs of each student class, utilizing evidence-based teaching practices while allowing the opportunity for mentorship, interclass connectedness, and refinement of the tutor's teaching skills. In this paper, we describe the creation of CLC, its goals, leadership and curricular structure, and its various benefits, challenges, and limitations.

## Introduction

In recent years, there has been increased incorporation of peer-assisted learning (PAL) modalities in medical education, a movement further advanced by the shift to virtual instruction stipulated by the coronavirus disease 2019 (COVID-19) pandemic.^1–5^ Many medical schools have moved from mandatory attendance for in-person lectures to virtual platforms, namely external resources such as video content (eg, Sketchy Medical) and question banks (eg, UWorld) dedicated to helping students pass their licensing exams.^6–8^ This change may have reduced faculty-student interactions, as students prioritized third-party resources over school-provided lectures.^9,10^ This paper will discuss the model of near-peer tutoring utilized in medical education, in which a senior student serves as an instructor to a junior student.

The University of California, Irvine, School of Medicine (UCISOM) emphasizes the innovation of novel teaching strategies to improve student learning through its mission: “Discover, Teach, Heal.” To ensure the development of scholarly physicians, a solid foundation of preclerkship knowledge is necessary. Traditionally, preclinical instruction comprises lectures taught by research and clinical faculty. In recent years, students have adopted a shift toward PAL.^4,10^ Originally developed for the elementary curriculum, PAL strategy programs allow peers to supplement classroom instruction.^11^ Outcomes associated with PAL programs have included improvements in academic domains, as well as tailoring differentiated instruction for the learning levels of students.^12^

PAL modalities have raised considerable interest in medical programs, as they can help institutions conserve resource measures, including faculty time and compensation, by allowing students to help close content gaps.^5,12,13^ Additionally, peer-assisted teaching provides students with the opportunity to practice teaching and assessment.^3,4,14^

The increased popularity of near-peer tutoring can be attributed to its efficiency, favorable outcomes, and positive student feedback.^15–20^ For example, peer tutoring is associated with affective support in which students feel they are treated as members of a single community within a warm and inclusive learning environment.^18,21^ Shenoy and Petersen^22^ built upon this idea and emphasized a main aspect of student benefit, social and cognitive congruence. Socially the tutees felt more connected and willing to divulge their feelings because of a shared understanding of the difficulties of medical school while cognitively they were able to glean knowledge for how to learn and integrate information at the medical student level.^22^ Along with affective support, near-peer tutoring has also improved tutee's examination scores and course grades.^22^ Additionally, as medical students advance in their education, near-peer tutoring has provided unique training opportunities for upper-class-standing students to refine their teaching skills.^22^ Trainees eventually become attendings responsible for educating younger trainees and their patients. As such, through participation in near-peer tutoring, the tutee and the tutor reap numerous benefits for their short- and long-term education and career, including the tutor refining their choice of career.^22–24^

At UCISOM, faculty and administration have appreciated the importance of near-peer teaching. In 2018, the anatomy course hired second-year medical students (MS2s) to host review sessions for first-year medical students (MS1s), with the physiology course shortly following suit as a separate program. These programs improved student performance, eased the medical school transition, and alleviated student anxiety.^25,26^ In 2020, UCISOM created the Collaborative Learning Communities (CLC) with Medical Students as Teachers (MSAT) program to facilitate a community learning environment, enhance student learning, and consolidate multiple educational services into 1 structure. Since its inception, CLC has expanded services to support first, second, and third-year (MS3) medical students.

## Methods

### Development of CLC

With the essential support and collaboration between faculty and medical education administration at UCISOM, medical students helped develop CLC in recognition that upper-class students could positively contribute to their peers’ education. Under CLC, UCISOM unified all tutors into 1 program, compensated them equally, and provided professional educational training to prepare staff for tutoring.

All resources utilized within CLC have been created by students, for students, with input from faculty and administered by the Department of Academic Support. For example, the MS2 program utilizes board-style question (BSQ) reviews where questions are adapted from coursework, textbooks, and online resources. Then, tutors create explanations for the correct answer and each wrong answer. Specifically, tutors regularly meet with faculty to review learning objectives and ensure the accuracy of the prepared materials. Meetings range from the tutor teaching that topic for the upcoming exam (eg, cardiovascular physiology) or the Curriculum Specialists and Program Directors to discuss items students are struggling with to ensure they are emphasized in the review sessions. Learning materials are reviewed by the Curriculum Specialists, Program Directors, and Director of Academic Support Services. Faculty meetings and review of materials occur sporadically and as requested by either the faculty or MSAT. Five hours of summer workshops help MSATs learn evidence-based pedagogical techniques and learning strategies from the Director of Academic Support Services to ensure consistency and effectiveness in their teaching process. The CLC program initially began as a pilot study for MS1s with 18 MSATs. This program was extremely well received by students and administration, which CLC has since expanded to include funding for 70 MSATs with content spanning the first, second, and third-year curriculum.

### Leadership Structure

**Figure 1.**
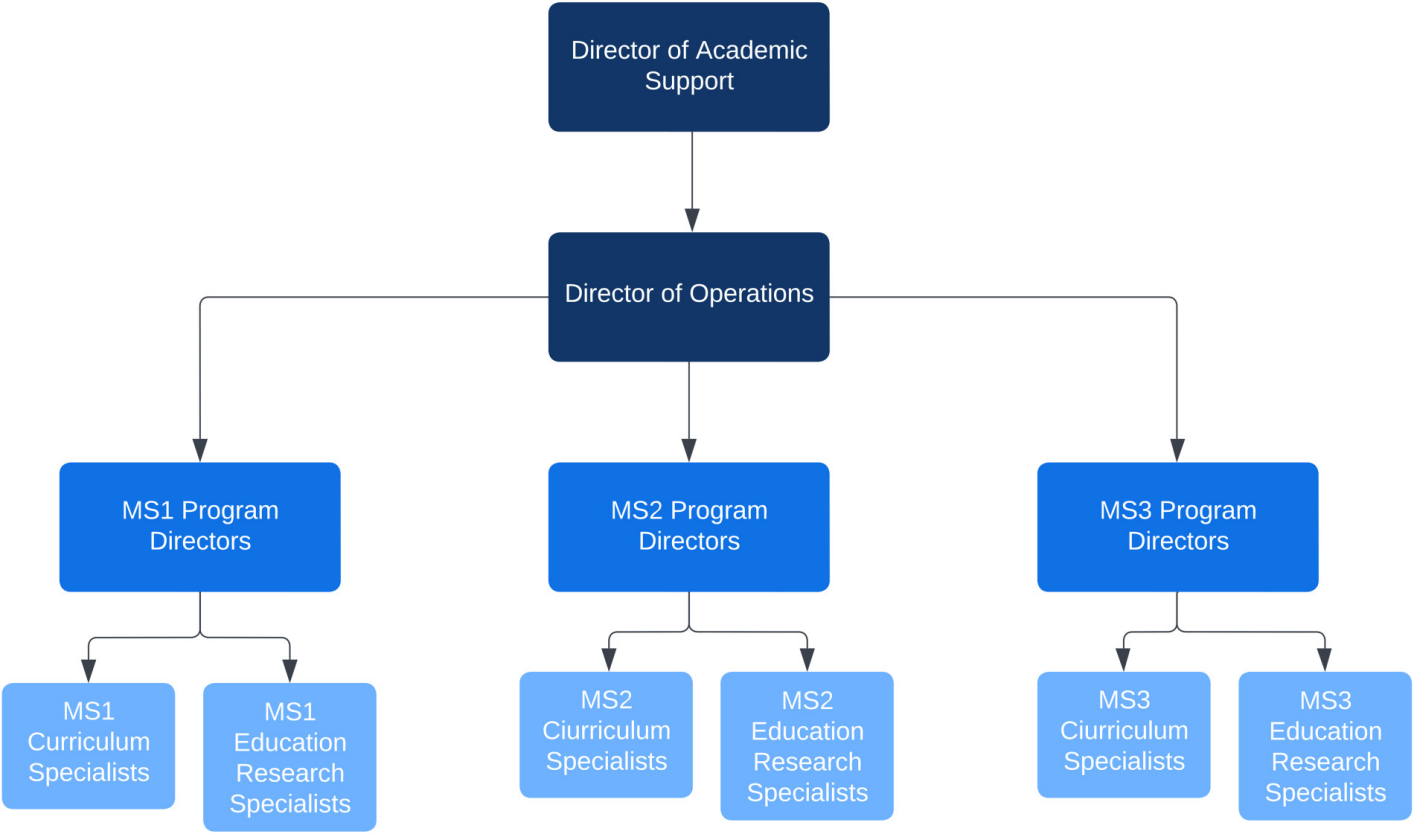
Leadership structure.

The leadership structure of CLC includes: A Director of Academic Support Services (staff administrator) who oversees and refines the CLC program based on student, staff, and faculty input, as well as leads the evaluation and research of the program, a Director of Operations (student, traditionally a third, fourth, or leave of absence student) who provides support in the implementation of the CLC-MSAT program by overseeing the day-to-day administrative operations of the program, MS1/MS2/MS3 Program Directors (2 students per program, 6 total) who assume leadership and responsibility for developing, coordinating, and implementing the CLC-MSAT program with excellence as well as refine program vision, mission, and strategy, Curriculum Specialists (2 students per program, 6 total) who develop instructional materials and curate resources to support instructional leadership for case-based and/or other learning sessions, Education Research Specialists (1 student per program, 3 total) who work collaboratively with the CLC team to facilitate education research projects and support systematic collection and analysis of data, a Social Media Specialist (1 student total) who manages the Instagram account of CLC to facilitate an online presence with information distribution, along with individual MSATs for each school year who plan, prepare, and deliver lesson plans to create active learning using evidence-based pedagogical methods to students (Figure 1). The responsibilities of each position ensure the program's continued excellence while maintaining communication and interconnectedness from all program levels. Above all, MSATs are consistently encouraged to put their own educational endeavors first, especially as their time commitment fluctuates heavily throughout the year. As dictated by university policy, all student positions are limited to 10 working hours per week.

CLC recruitment occurs annually, with applications sent out to first through third-year medical students in early Spring. In conjunction with the Director of Academic Support Services, a team of twenty current MSATs review and score applications following a uniform rubric with the applicant's identifying information redacted to preserve integrity in evaluation. The rubric measures responses from 3 open-ended items on the survey related to the applicant's past teaching experience, how the program aligns with career goals, and how the applicant will contribute to inclusive excellence in teaching. Following this process, the Director of Academic Support Services reviews all scores and applications, then offers employment to approximately 75% of the top applicants. While final employment considerations account for some measures of the applicant's academic performance, the decision to not extend an offer to a student who received a high overall application score would typically be reserved for those who meet extreme underperformance criteria at UCISOM, such as the risk of dismissal. In 4 cycles of recruitment, we have yet to encounter this scenario. While most of our team members have been academically successful, MSATs range in their track record within the medical school. For example, several MSATs have utilized CLC individual tutoring and support through the Learning Specialists to remediate performance at some point in their academic journey. Thus, MSATs are selected holistically rather than solely based on academic performance, as open-ended responses on past teaching experience and the ability to serve students of diverse learning needs are the foremost priorities for recruitment.

### MS1 Program

MS2s lead the CLC program for MS1s and emphasize the adoption of successful learning strategies and habits in the first months of medical school to facilitate a smooth transition from undergraduate learning. Students are introduced to CLC during their MS1 orientation when they meet the school's Learning Specialists, CLC leadership, and MSATs to learn about the MS1 program structure. The CLC programming for MS1s includes large-group preexam review sessions open to all MS1s (114 students), small-group bimonthly content review sessions (students opt into these sessions in the weeks prior to the first session), individual tutoring, and various workshops. All CLC programming is optional and session materials are shared widely within the medical school to encourage the utilization of these resources.

There are 13 two-hour, large-group preexam review sessions in which MSATs present high-yield concepts from lectures for the Physiology, Histology, and Neuroscience courses. There are also seven 1-hour, small-group sessions in which MSATs lead approximately 10 to 15 students through prepared worksheets, emphasizing active learning strategies in conjunction with navigating clinical vignettes. For example, students are encouraged to reorganize critical information from the vignette in a graphic organizer, provide supporting and refuting evidence for each answer choice, and connect new information learned from each session to their existing concept maps. MSATs receive training in backward planning, appreciative inquiry tutoring, and evidence-based learning strategies from the Director of Academic Support Services upon hire to enhance consistency in pedagogy. Individual tutoring is provided by student request and consists of once-weekly sessions between 1 student and 1 MSAT. Although the number of individual tutoring requests and length spent utilizing the service varies yearly, typically, between 5 and 15 students use this service at any given point during the academic year. Lastly, MSATs host workshops covering study strategies, outside learning resources, mentorship, and extracurricular activities. Most MS1 CLC activities are currently held via Zoom, though students and MSATs reserve the flexibility to conduct their sessions in person.

The MS1 CLC program also incorporates anatomy tutoring. At UCISOM, MS1s learn human anatomy through didactic lectures and lab dissections. Several exams are administered as “practicals,” which incorporate identifying anatomical structures on human dissections in the labs. To facilitate learning and better prepare students for these unique exams, CLC has implemented additional modalities of review for anatomy. These include more than 10 four-hour laboratory review sessions, 4 practice practical exams simulating test day conditions, and lecture reviews. All anatomy tutors must partake in a meeting in which the anatomy course director outlines how to effectively tutor anatomy and subsequently renew safety training modules before returning to the laboratory.

### MS2 Program

MS3s lead the CLC program for MS2s. The program provides 2 primary services—pre-exam and BSQ reviews which cover the Pathology, Pharmacology, Microbiology, and Behavioral Science courses. BSQ reviews are held 9 times in sync with exam reviews. Students are familiarized with answering questions like those seen on the United States Medical Licensing Examination (USMLE) Step 1. There are currently 10 BSQ groups that meet throughout the year with an optional drop-in group for students not assigned to a group longitudinally. There are no more than 10 students per BSQ group. BSQ reviews cover 5 to 6 questions per subject, allowing students time to read, work through, and select answers individually. MSATs emphasize important information from the question stem, pose leading questions, and encourage students to identify helpful information to find the answer and eliminate incorrect choices. This method emphasizes active learning, allows students to think comprehensively, and instills valuable test-taking strategies. Once the students reach the correct answer, MSATs will present question-relevant content review slides outlining high-yield topics and other relevant information for the subject.

Three to 4 MSATs conduct preexam reviews in large-group format (n = 104), each presenting high-yield information for a specific subject. Rather than comprehensive course reviews, these sessions are intended to distill exam content into a new, memorable format, with the expectation that students have comprehensive understanding of class material before the sessions. The aforementioned BSQ sessions currently take place immediately following the exam review. MSATs also host reviews for second-year National Board of Medical Examiners (NBME) “shelf” exams in Microbiology, Pharmacology, Pathology, and Behavioral Sciences. These sessions cover the entirety of the subject with content review and BSQs.

Additional workshops are held throughout the year, including sessions discussing study tools, strategies, outside resources, and USMLE Step 1 preparation. Individual and small-group tutoring is also available by request.

### MS3 Program

The CLC program for MS3s is directed and instructed by fourth-year medical students. At UCISOM, the MS3 curriculum consists of 7 core clinical clerkships—Internal Medicine (IM), Surgery, Obstetrics and Gynecology (OB/Gyn), Pediatrics, Psychiatry, Neurology, and Family Medicine (FM)—with students split into “rotation tracks.” MS3 CLC is divided into 7 concurrent programs corresponding to each core clerkship. These programs provide supplemental instruction and academic support for each clerkship throughout the year, with recurring curricular schedules every 4 (Neurology, FM), 6 (OB/Gyn, Pediatrics, Psychiatry), or 8 (IM, Surgery) weeks. Each program provides 4 primary services: an Introduction to the Rotation session, an Observed Structured Clinical Exam (OSCE) session, a review session for the NBME “shelf” exam (Shelf Review Session), and academic mentoring.

Two to 3 MSATs oversee each clerkship program for the entire academic year. These MSATs generate the instruction schedule for each rotation of students in their clerkship, create and edit the Shelf Review Session slides, conduct the Introduction to the Rotation, OSCE Q&A, and Shelf Review sessions, and serve as academic mentors for current clerkship students. With respect to the COVID-19 pandemic and to accommodate differences in schedules and locations, all MS3 sessions are currently held via Zoom.

Introduction to the Rotation sessions are 20 to 40 minutes in duration and held in the first week of each rotation. These sessions outline the timeline of CLC services, provide early guidance for students, and encourage proactive preparation for exams to foster student success. MSATs discuss study resources and strategies for the clerkship and make themselves available for students’ questions. OSCE sessions take place 1 week before each rotation's OSCE and provide a guided review of relevant clinical skills, while also allowing time for student questions. Content covered includes the format and timing of patient encounters, advice on exam preparation, expectations from evaluators, history-taking and physical exam review, and role-play through patient encounters. To maximize utility for learners, the timing and format of these sessions are designed to be flexible so that MSATs can accommodate students’ needs.

Shelf Review Sessions take place 1 to 2 weeks prior to each NBME. These sessions provide a guided review of high-yield topics, allow real-time feedback through practice questions, and provide additional study materials to learners. Students are presented with BSQs covering commonly tested topics and are given 1 to 2 minutes per question to read, consider answer choices, and select an answer. After this time, MSATs guide learners through each question and its answer choices, helping them identify pertinent details to guide them to the correct choice. MSATs then discuss the answer and review the core topic with preprepared review slides. After each Shelf Review Session, the presentation slides containing question answers, explanations, and content review slides are emailed to all learners in the rotation.

MSATs also serve as academic mentors for each cohort of rotating students by making themselves available for assistance and advice about the clerkship. Academic mentors give students multiple contacts throughout their rotation to encourage open communication and reduce time spent struggling before asking for assistance. Typically, this role involves fielding questions about study resources, study strategies, and how to prepare for or improve clinical duties.

Refer to Table 1 for a summary of the structure of each program year.

**Table 1.** Outlining Services Provided by CLC With Structural Similarities And Differences Denoted For Each Medical School Year.

**Program by year**	**MSAT year**	**Exam review sessions ** **n(h)**	**Small group sessions ** **n(h)**	**NBME exam review n(h**	**Courses included**	**Individual tutoring**	**Specific topic workshops**
**MS1**	MS2	13(2)	7(1)	N/A	Physiology, histology, neuroscience	Upon student request	- How to prepare a *Curriculum Vitae* - NBME Q&A and study tips and tricks - MS1 Intro to Anki - Study habits Q&A
**MS1 Anatomy**	MS2	10(3) laboratory	N/A	N/A	Anatomy	Upon student request	N/A
**MS2**	MS3 and LOA	9(1.5)	9(1)	4(2)	Pathology, pharmacology, microbiology, behavioral science	Upon student request	- Intro to AnKing and study schedule excel sheet - Intro to common resources - How long should Dedicated be? - Crafting a Dedicated schedule - How to tackle UWorld questions - Intro to Step 1 - What to expect on Step 1 test day
**MS3**	MS4 and LOA	N/A	N/A	7(2)	Internal medicine, surgery, obstetrics and gynecology, pediatrics, psychiatry, neurology, family medicine	Upon student request	- Crafting a step 2 Dedicated schedule - For each rotation course: - Introduction to the rotation session - Observed structured clinical exam session

Abbreviations: LOA, leave of absence: Including PhD, MBA, MPH, and Research year students; NBME, National Board of Medical Examine; CLC, Collaborative Learning Communities.

### Additional Support

UCISOM students with pervasive difficulties in medical school are encouraged to utilize CLC and Learning Specialist support. The Director of Academic Support Services also serves as 1 of the 2 UCISOM Learning Specialists. While there is no limit to the number of sessions a student may book with a Learning Specialist, most students request 5 or fewer sessions throughout their 4 years of medical school. In contrast, students with the greatest need often meet weekly for an hourly session. All services are offered at no cost to medical students and are supported by funding from the medical education department.

### Learning and Teaching Models

Since its inception, the CLC program has identified 3 priorities: easing student transition into medical school learning, helping students gain a comprehensive understanding of medical content, and cultivating a sense of belonging. Thus, CLC is structured to ensure dedicated support is available for students of all learning levels.

CLC Program uses the following theoretical models to support high-quality student-focused learning: Social cognitive learning theory, adult learning theory, and situated learning theory. Specifically, our program supports the reciprocal nature of learning which occurs in a social context, dependent on a learner-centered and safe environment.^27^ For example, the MSAT is the learning facilitator, providing enthusiasm and individualized guidance to students when interacting with complex content material. As a student's learning capacity increases, the MSAT removes scaffolding until the student feels proficient in the subject. This reciprocal nature of teaching and learning also benefits the MSAT, as they gain proficiency in the subject they are teaching and can explain, anticipate, and answer questions on challenging material.

As the hallmark of adult learning theory, our students are engaged in solving problems rather than rote memorization and regurgitation. Thus, our services ensure adult learners have opportunities to critically apply their knowledge to content directly relevant to their future practice.^28^ These include navigating clinical vignettes of patient presentations, which involve determining a diagnosis and providing recommended next steps in treatment and prognosis. Our active learning experiences strive to uphold the utility of gaining knowledge when embedded within authentic contexts of practice.^29^ Our investment in providing near-peer instruction using authentic learning experiences helps support the situated learning theory's approach highlighting the importance of the social dimension in teaching. Specifically, the opportunity to discuss clinical cases between a tutor and tutee helps situate their knowledge within a clinical context, providing a social choreography in determining and justifying a judgment between the 2 parties.^30^

Our educational partners are responsible for practicing humility, applying a growth mindset, and participating in continuous self-reflection to ensure we meet the changing needs of our students. As educational partners within the CLC program, we strive to use our diverse assets to bridge performance gaps between students who may have been traditionally divided.

### Duration and Nature

Since CLC's creation in 2020, student surveys have been and continue to be conducted 3 times per academic year for each program. Beginning in 2022, extensive attendance data has been collected for the MS1 program and will be collected for the MS2 and MS3 programs in the 2023 academic year. The total period of study for survey data has been 2 years and 5 months. In the following paragraphs, results from the surveys, attendance data, and data from the Office of Academic Support Services will be presented.

## Results

CLC continues to evolve in response to student feedback and annual satisfaction surveys. Due to the unique challenges of implementing multiple levels of tutoring, CLC has deliberately phased into additional training programs on an annual basis. The MS1 program was implemented in 2020, MS2 in 2021, and MS3 in 2022. By implementing new programs in phases and conducting annual student satisfaction surveys for each program, CLC has established a solid foundation in preparation to expand and refine service offerings on an annual basis. Yearly changes have included a complete revamping of the biweekly small-group worksheets for the MS1 program and a shift in the focus of MS2 exam reviews from comprehensiveness to organized and memorable information synthesis. Due to the COVID-19 pandemic, there also has been an adjustment in learning modalities used by MSATs. While the program began with primarily in-person instruction, almost all CLC sessions are now held remotely via Zoom video conferencing. This shift was a necessary consequence of needing to continue providing academic support amidst the pandemic which has now developed into a preferred content delivery method by most students and tutors. We have found that this modality increases attendance and student comfort while allowing for polling and anonymous learner messaging.

Before the onset of CLC, meeting with a Learning Specialist was typically viewed as a service reserved solely for extreme underperformance. However, MSATs have helped change the stigma surrounding seeking assistance before struggling. In comparison to 2020, when approximately 10% of first, second, and third-year struggling medical students met with a UCISOM Learning Specialist for at least 1 session, by 2023, nearly 30% of students on a spectrum of academic performance have met with a Learning Specialist. While other external factors such as the COVID-19 pandemic may have influenced students’ receptivity to booking an appointment with a Learning Specialist, informal dialogue seems to suggest the importance of MSAT influence on students. For example, when asked at the beginning of a session of what brought the student to meet with a Learning Specialist today, many students have indicated their MSAT recommended they meet with a Learning Specialist to discuss evidence-based learning strategies. Thus, it is difficult to ignore the impact MSATs have had on helping increase the utilization of Learning Specialist support in conjunction with CLC content support. Together, these entities are now seen as important resources of our university culture rather than intended solely for underperformance.

Based on a 1 to 4 Likert scale, an item from our student satisfaction surveys for the 2021 to 2022 academic year for the MS1 program (n = 37, 36% response rate) indicated 92% of students “Strongly Agreed or Agreed” their learning was enhanced by CLC sessions, which led to positive results towards personal goals after the session. This is in comparison to the MS2 program (n = 19, 18% response rate) in which 84% of students “Strongly Agreed or Agreed” their learning was enhanced by CLC sessions, which led to positive results towards personal goals after the session. For the MS3 program (n = 12, 12% response rate), 92% indicated they “Strongly Agreed or Agreed” with this same item.

Prominent themes for responses within open-ended items soliciting feedback for program improvement for the MS1 program included: increasing the frequency of CLC sessions, MSATs using time more efficiently, and focusing more on board-style questions than the review of content. MS2 program feedback was generally administrative, such as requests for additional break time during learning and hosting sessions earlier in the evening. MS3 program feedback encouraged MSATs to ask students for feedback at the halfway point of a clerkship for topics they continue to have difficulty understanding, as well as to increase the number of email reminders for CLC sessions. Results from these surveys are shared with all stakeholders of CLC, including faculty administration and MSATs. The student directors of each CLC program are responsible for determining and communicating to their team on how specific feedback items can be addressed for the unique operations of their program. The Director of Academic Support Services then consults with each program director about the status of adopting new feedback, typically monthly.

Along with survey data, attendance data were also collected. For the MS1 program during the academic year 2022 to 2023, 107 students (94%) enrolled in the small-group sessions. Throughout the 13 large-group pre-exam review sessions, the average number of attendees was 90 (79%; min = 56, max = 108), and students attended an average of 84.18% of the session duration (as calculated by taking the average number of minutes each student attended a particular session divided by the total session duration then produced as an average for all 13 sessions). When analyzing whether an MS1 partook in either the small-group, large-group, or both, we recorded that 100% of MS1 s in 2022–2023 utilized CLC services. For the MS2 program, attendance for the BSQ reviews was roughly 80 students (77%), and pre-exam reviews were between 30 and 90 students (29%-87%). Between 31 and 52 students (30%-50%) attended each of the MS3 program sessions. Outside of general enrollment, attendance for the MS1 small-groups and MS2 BSQ groups was not substantially recorded nor analyzed.

In addition to increased, proactive, help-seeking behaviors from students of all learning levels, another student outcome associated with CLC includes increased shelf exam performance. For example, with the initiation of our first year of MS3 services in December 2022, UCISOM experienced a 100% shelf examination pass rate for 1 cycle of shelf exams. This milestone took place for the first time at our university, in which faculty largely credited the efforts of MSATs for preparing students for each of their clerkships.

The program's continued growth is further reflected in the increase in the number of tutors due to increased demand and interest. In the 2020 to 2021 pilot program, 18 tutors were hired. For the 2021 to 2022 program, we had a team of 39 MSATs. Now in our 2022–2023 program, the CLC team consists of 48 MSATs. For the 2023–2024 academic year, CLC intends to grow to a team of 70 MSATs. Additionally, in our most recent recruitment cycle, we received applications from nearly 40% of matriculated students at UCISOM. This is in comparison to the 2020 pilot program in which roughly 25% of UCISOM students applied to be an MSAT. The increase in MSATs is the result of additional funding provided by medical administration in response to an outpouring of support and increased enrollment associated with the program. MSAT positions have become highly sought-after, in which the number of applications greatly exceeds the positions available for any academic year.

## Discussion

CLC is committed to utilizing student feedback to improve program services. Specifically, students are invited to provide feedback on the program 3 times per year through anonymous surveys. The MS1 program typically has the highest survey response rates, whereas the MS3 program has the lowest of the 3 programs (36% compared to 12%, respectively). Three students are randomly selected for a gift card during each survey administration. Students from all programs generally agree that CLC has enhanced their learning. Through surveys, embracing student concerns, and monitoring the ever-changing medical education landscape, the CLC program ensures it remains proactive and receptive to change.

While attendance rates vary between each program's sessions, the MS1 program continuously engages with the highest number of students of all 3 programs (at an astounding rate of 100% of the current MS1 class). A significant focus of the MS1 program is to destigmatize medical school tutoring by framing it as a resource that most, if not all, students will utilize, regardless of academic performance. This is compared to traditional perspectives regarding tutoring (eg, 1 only needs a tutor if they are struggling with a subject). Attendance rates decrease in the second year as students gain confidence in their study strategies and shift toward third-party resources. The third-year curriculum takes a relevant change focusing on shelf reviews and adopting more varied curricular schedules. Furthermore, it does not include small-group learning as learners become more experienced and less reliant on CLC services. For all 3 programs, content is shared widely throughout each class level so those who do not, or are not able to, attend can still utilize the prepared resources. This growth of CLC has been made possible through the repeated success of high student enrollment, tutor interest, and an effective expansion of services, as measured by student satisfaction surveys and UCISOM administration financial support.

Overall, the high level of involvement in academic tutoring may have helped reduce the stigma of participating in educational support services while encouraging help-seeking behaviors for our students. Similarly, each year, more faculty contribute to the CLC program, such as providing study guides, discussing difficult content areas, and consistently collaborating with tutors to ensure the programs run efficiently and effectively. CLC also focuses on creating and running new topic-focused workshops for each class level. For example, students in the MS1 program are offered workshops on how to craft a *Curriculum Vitae*, prepare for NBME exams, and focus on wellness in conjunction with the rigors of medical school. With these sessions, CLC reaffirms its commitment to academic support, mentorship, professional development, and encourages students to seek out assistance proactively.

### Lessons Learned

At UCISOM, we have successfully created and facilitated a longitudinal, near-peer teaching model where the tutor is 1 academic year more advanced than the students being tutored. This model has aided overall medical student learning through improvements in student academic performance, mentorship, professional development, and the perception of asking for support. The success and effectiveness of our near-peer teaching model rely heavily on general medical students’ preference and willingness to learn through peer tutoring as well as the quality of tutors and curriculum material.^31^ Both of these were achieved through our CLC program, along with the added benefit of engaging senior MSAT, providing them with the opportunity to become confident in assuming a teaching role. The positive benefits of having a near-peer teaching model have been reported for both the student teachers and the mentee learners.^32^

The majority of our CLC sessions are separated into optimal teaching group sizes, with 1 peer tutor responsible for 7 students. This allows for dedicated attention to be appropriately distributed to all students in the program. Additionally, CLC utilizes the integrated teaching of BSQs with pure content review, which provides students the added benefit of clarifying any concepts they may not understand fully before diving into the technique of how to answer BSQs. Throughout the program, we provide students with structured slides and question stems, which present medical knowledge and content while guiding students through analytical thought processes on parsing important information from a clinical case.

The success of our program could only be achieved with faculty support and collaboration from the medical school administration. Individual professors provide their teaching material for tutors to review, course directors provide helpful guiding information for in-class and board exams, and the administration provides funding for tutors’ compensation. Medical school is demanding, and compensating students while providing opportunities to gain leadership and tutoring experience has made CLC a highly sought-after after program. MSATs treat this position as a formal job rather than an extracurricular activity, pushing them to take accountability for their students’ success. As demonstrated through the annual evolution and expansion of the CLC program, tutors are dedicated to enhancing the learning environment for medical students at UCISOM.

After witnessing the benefits of CLC-MSAT, we call on other medical schools to implement similar near-peer tutoring services, as we have outlined, and adjust for their student needs. Building upon peer tutoring programs that already exist at other medical schools, our program is structured to address the academic support needs of students of all learning levels at our university, facilitate healthy mentorship relationships among medical students, and provide students with authentic teaching experiences. Together, these opportunities may help positively contribute to the conception of our future academic clinicians.

### Obstacles

Creating and sustaining CLC with excellence has led us to several obstacles. First, administrative and faculty support is required for the success of this program. We have experienced several communication challenges between some MSATs and faculty in response to curricular changes that were not widely shared with the CLC team. Another difficulty has been with scheduling tutoring sessions. The Program Directors establish a consistent schedule (eg, small-group sessions are always Wednesday evenings) and disseminate that schedule during the first week of instruction in August. However, overlapping events and schedule changes can cause confusion and deviation from the original plans.

Time is another major obstacle. MSATs are medical students first and employees second. Thus, we have experienced occasional changes to program execution due to the time constraints of our MSATs. The program is fortunate to be run by students and for students, in such a way that it can be maximally effective despite these inevitable obstacles.

### Limitations

Several limitations exist for the CLC program. First, despite training and workshops from leaders with advanced degrees in education, there is difficulty in standardizing MSAT teaching methods. Given the sheer number of staff and autonomy MSATs are provided in teaching, there is a spectrum of teaching practices. While this can be positive, we also acknowledge the diversity in teaching styles may suit some students better than others. Second, medical students are inherently busy and may be unable to cater to each student's learning preferences. Specifically, MS3 MSATs must manage their tutoring duties with both long hours at the hospital and studying for their own coursework.

Next, attendance data are not a standard collected measure for all 3 programs. This has limited some of the conclusions presented in the Results and Discussion. In a similar manner, our adapted survey of student attitudes and satisfaction has not been validated nor pilot tested. However, we are confident our survey measure as it was adapted from the Online Student Connectedness Survey, a thoroughly tested, valid, and reliable survey (α=0.98).^33^ Lastly, despite positive intentions, our university is not fully immune to the stigma surrounding tutoring being reserved for struggling students. However, at UCISOM, students are exposed to our learning services from their first day of medical school, in which participation in CLC for students of all learning levels has largely become part of the UCISOM culture.

### Future Directions

As the CLC program continues to evolve each year, it also seeks to improve student wellness, student-to-student career advising (eg, *Curriculum Vitae* support, projects and roles students have taken on to aid them in their residency application, networking options), and assistance for those in gap years (eg, research, leave of absence, masters years). For the 2023 to 2024 academic year, CLC will fall under a new program organization, ZotUnity, which will unify our academic support services with existing university wellness programs. This program intends to continue the expansion of CLC while applying its successful groundwork to student wellness initiatives. Surveys will be conducted in a similar manner as they are now, but will begin collecting data on student wellness directly. Thus, a concerted effort is being made to adapt CLC's successful model as a blanket template for other programs at the SOM, such as peer mentorship and career advising.

## Conclusion

The CLC program has continuously grown, innovated, and become more successful at serving the diverse learning needs of students. Positive outcomes associated with CLC have included: an average of 89% of students across the 3 programs strongly agree or agree CLC has enhanced their learning, a 20% increase in the utilization of Learning Specialists, and the first 100% shelf examination pass rate at UCISOM with the onset of our MS3 pilot program. Innovations have included novel exam workshops, biweekly small-group worksheet sessions, comprehensive anatomy reviews, and OSCE and shelf review sessions for third-year students. CLC continues to innovate peer-tutoring, demonstrated by the successful adaptation from an entirely in-person to a virtual model through video conferencing software. All CLC programs and events continuously garner positive feedback, with services boasting an impressive enrollment rate of over 95%. CLC strives to ensure MSATs are supported both professionally and financially, as they provide high-quality learning services to our students. In an effort to cultivate responsive academic clinicians, we encourage all medical schools to adopt similar peer tutoring services for their medical students.
